# Multigenerational fitness outcomes of double-brooding: a 30-year study of a migratory songbird

**DOI:** 10.1093/beheco/araf040

**Published:** 2025-05-04

**Authors:** Hayley A Spina, Amy E M Newman, Nathaniel T Wheelwright, Daniel J Mennill, Stéphanie M Doucet, Joseph B Burant, Sarah L Dobney, Sarah D Mueller, Greg W Mitchell, D Ryan Norris

**Affiliations:** Department of Integrative Biology, University of Guelph, 50 Stone Rd E, Guelph, Ontario, Canada, N1G 2W1; Department of Integrative Biology, University of Guelph, 50 Stone Rd E, Guelph, Ontario, Canada, N1G 2W1; Department of Biology, Bowdoin College, 255 Maine St, Brunswick, ME 04011, USA; Department of Integrative Biology, University of Windsor, 401 Sunset Ave, Windsor, Ontario, Canada, N9B 3P4; Department of Integrative Biology, University of Windsor, 401 Sunset Ave, Windsor, Ontario, Canada, N9B 3P4; Department of Integrative Biology, University of Guelph, 50 Stone Rd E, Guelph, Ontario, Canada, N1G 2W1; Department of Animal Ecology, Netherlands Institute of Ecology, Droevendaalsesteeg 10, 6708 PB, Wageningen, the Netherlands; Department of Integrative Biology, University of Windsor, 401 Sunset Ave, Windsor, Ontario, Canada, N9B 3P4; Department of Integrative Biology, University of Guelph, 50 Stone Rd E, Guelph, Ontario, Canada, N1G 2W1; Wildlife Research Division, Environment and Climate Change Canada, Ottawa, Ontario, Canada; Department of Integrative Biology, University of Guelph, 50 Stone Rd E, Guelph, Ontario, Canada, N1G 2W1

**Keywords:** double-brood, lifetime fitness, recruitment, multi-brood, breeding strategy, survival, multigenerational analysis

## Abstract

In birds, rearing multiple broods per season can substantially increase the annual number of fledglings produced. However, the contribution of double-brooding to lifetime fitness is unclear because the number of recruits arising from single- and double-brooded females is rarely measured. Poor estimates of fitness also make it challenging to document potential trade-offs between double-brooding and survival or future reproductive output. To understand the contribution of double-brooding to lifetime fitness and whether double-brooding was associated with life-history trade-offs, we used 30 years of reproductive data on female Savannah sparrows (*Passerculus sandwichensis*) breeding on Kent Island, New Brunswick. Estimates of fitness included an analysis of recruitment of both F1 (first generation) and F2 (second generation) offspring from females that did and did not raise a second brood. We detected no net costs of double-brooding. Double-brooded females had higher annual apparent survival rates than single-brooded females and F1 offspring from first broods of double-brooded females were more likely to recruit into the population than F1 offspring from single-brooded females. Double-brooding also improved lifetime fitness. Recruitment of F1 offspring was positively related to the number of seasons that a female double-brooded and, as a result, there was a higher number of F2 recruits from F1 offspring arising from double-brooded females than from F1 offspring arising from single-brooded females. Our results provide strong evidence that double-brooding is a beneficial reproductive strategy for Savannah sparrows and suggests that double-brooding females are likely high-quality individuals capable of rearing two broods a season with no net fitness costs.

## Introduction

Life-history theory predicts that individuals allocate limited resources across activities in such a way that maximizes fitness and that investment into current reproduction may limit investment into future reproduction or survival (Williams 1966; Stearns 1976, Stearns 1989; Martin 1987). However, in birds, where clutch size is an important indicator of reproductive investment, strong trade-offs between the number of eggs produced and survival or future reproductive success are not often observed in response to natural variation in clutch size (Tuomi 1990; Godfray et al. 1991). One reason for the absence of these trade-offs may be because variation in clutch size is typically low for songbirds (Billerman et al., 2022) and may not reflect variation in the total energetic investment in reproductive output. Instead, in populations where it is possible to successfully rear two broods in a season, the primary currency of the survival-reproduction trade-off could be the number of broods reared per season (Martin 1987). By increasing energetic investment in reproduction through the production of multiple broods, females can substantially increase their reproductive output (Den Boer-Hazewinkel 1987; Geupel and DeSante 1990; Husby et al. 2009; Bulluck et al. 2013; Townsend et al. 2013; Agnew et al. 2014; Cornell and Williams 2016; Woodworth et al. 2017). However, in females, increasing investment in current reproductive efforts may be constrained by a cost to self-maintenance, future survival, or future reproductive effort (Verhulst 1998).

Knowledge of the benefits and potential costs of double-brooding to individuals and their offspring is necessary to quanitfy the value of double-brooding and address whether double-brooding truly increases fitness. Costs of double-brooding to survival have generally not been observed in correlational studies comparing the annual survival or lifespan of single- versus double-brooded females (Geupel and DeSante 1990; Evans Ogden and Stutchbury 1996; Hario 1997; Husby et al. 2009; Carro et al. 2014; Zabala et al. 2020). However, an experiment demonstrated higher survival in females that had their second broods removed the year before than females that fledged their second broods (Verhulst 1998). Experimental studies are more likely to detect costs of double-brooding because positive correlations between life-history traits (e.g. double-brooding and survival) are expected when there is variation in individual quality within a population (van Noordwijk and de Jong 1986). Thus, it is possible that past studies have not found that double-brooded parents trade-off survival for reproduction primarily because most have been correlational in nature.

An alternative reason that past studies have not detected survival costs to double-brooding could be that double-brooding poses costs to future reproductive success instead of survival. Few studies have investigated this possibility, likely because of low return rates and difficulties in repeatedly finding nests belonging to the same individuals across years (Cornell and Williams 2016). Although several studies have shown that double-brooding increases lifetime fledgling production (Carro et al. 2014; Hoffmann et al. 2015; Johns et al. 2018; Zabala et al. 2020), double-brooded females might trade off offspring quality for quantity. For example, double-brooded parents may reduce post-fledging care of the first brood to invest in the initiation of a second brood (Geupel and DeSante 1990; Morrison 1998; Kloskowski 2001; Grüebler and Naef-Daenzer 2008a, 2008b) and second-brood nestlings can be in poorer health or body condition than first-brood nestlings (Wilson et al. 1987; Aguon and Conant 1994; Dubiec and Cichoñ 2001; Antonov and Atanasova 2003; Muriel et al. 2015; Cornell and Williams 2017), which may result in lower between-year apparent survival. Specifically, second broods have been shown to have lower recruitment in coal tits (*Parus ater*; Dietrich et al. 2003; Schmoll et al. 2003), house wrens (*Troglodytes aedon*; Hodges et al. 2015), and crested caracaras (*Caracara plancus*; Morrison 1998). In stitchbirds (*Notiomystis cincta*; Low et al. 2007), recruitment did not differ between broods. However, only the crested caracara study split the “first brood” group between offspring hatched in nests of single-brooded females and offspring hatched in nests of double-brooded females (Morrison 1998). Thus, the question of whether first-brood offspring of single- versus double-brooders differ in recruitment remains largely unstudied. Further, relatively few studies have examined lifetime recruitment in single- versus double-brooders (however, see Hoffmann et al. 2015; Zabala et al. 2020; Zając et al. 2015).

Double-brooding might also have multigenerational consequences, which could reflect ‘hidden’ costs of double brooding not apparent within one generation. In great tits (*Parus major*), when second broods were removed before hatching, first-brood recruits had higher reproductive success in their first breeding season than recruits from parents that raised a second brood, suggesting a multigenerational cost of second broods to first-brood offspring (Verhulst et al. 1997). How much second-brood offspring contribute to the fitness of double-brooders also remains in question, given that first-brood offspring can have higher reproductive output in their first breeding season than second-brood offspring (Zabala et al. 2020; but see Schmoll et al. 2003). Whether first-and second-brood recruits differ in lifetime fledgling production has only been investigated in one study, which found no differences in lifetime fledgling production (Zabala et al. 2020). No studies have compared lifetime recruit production in the second generation (F2); that is, recruits produced by first generation (F1) offspring hatched from single- versus double-brooded females. Given the variation in findings and lack of lifetime studies investigating multigenerational consequences of double-brooding, it remains unclear whether double-brooding carries a substantial cost to F2 offspring fitness.

Given that double-brooding requires substantial energetic investment, costs of double-brooding could be more likely to emerge when double-brooding is combined with other energetically challenging situations. Several situations experienced during the breeding season could be energetically challenging for female songbirds, such as being mated to a polygynous male, breeding during high intra-specific density, breeding for the first time, or rearing a large number of first-brood fledglings. Polygynous males may contribute less to parental care (Alatalo et al. 1982; Mueller et al. 2025), such that females mated to polygynous males may invest more into breeding than females mated to monogamous males. High population densities can increase predation and intra-species competition (Sillett et al. 2004; Woodworth et al. 2017), with higher investment required toward predator evasion, mate guarding, or resource acquisition. Females breeding for the first time may have the additional challenges of learning to avoid predation and find resources (Bildstein 1984; Woodworth et al. 2017). No studies have explored the combined impact of double-brooding and these energetically challenging situations on fitness.

Savannah sparrows (*Passerculus sandwichensis*) are migratory songbirds that may double-brood following the fledging of one or more offspring from their first brood, resulting in increased annual fledgling production when successful (Woodworth et al. 2017). On Kent Island, New Brunswick, Canada, a population of Savannah sparrows has been monitored since 1987 (Wheelwright and Mauck 1998). In this population, individuals demonstrate high philopatry and short between-breeding-season dispersal distances (mean = 31.8 m; Wheelwright and Mauck 1998) relative to territory sizes (mean territory diameter = 38 m; Wheelwright and Mauck 1998), making it possible to estimate lifetime fitness with a relatively high degree of accuracy (Mitchell et al. 2012; Burant et al. 2022). Previous research on Kent Island has demonstrated that Savannah sparrows fledging offspring late in the breeding season had higher overwinter survival than parents fledging offspring earlier and that the number of F1 offspring fledged did not impact their apparent annual survival (Mitchell et al. 2012). However, F1 offspring that fledged later in the season had reduced apparent annual survival (Mitchell et al. 2011). If late fledging offspring were more likely to be from the second broods of double-brooded females, this finding could suggest net costs of double-brooding to offspring survival but not maternal survival (Verboven and Verhulst 1996; O’Brien and Dawson 2013). However, late fledging offspring may not be offspring from second broods but from replacement nests of the first brood attempt (Wheelwright and Rising 2020), so it remains uncertain whether there are survival costs of double-brooding.

We analyzed 30 years of data to examine the benefits and potential costs of double-brooding on multiple components of fitness. Long-term monitoring of Savannah sparrows on Kent Island provides a unique opportunity to investigate lifetime fitness outcomes of double-brooding in females and their offspring, including the novel exploration of whether being reared by a double-brooded mother impacts lifetime F2 recruit production. Specifically, we measured annual apparent survival and number of F1 offspring fledged in single- and double-brooded females. We also measured whether lifetime F1 recruit production was influenced by the number of seasons that females were double-brooded. Additionally, we measured recruitment, lifetime F2 fledgling production, and F2 recruit production in F1 offspring of single- and double-brooders. In doing so, we provide a comprehensive analysis of the benefits and costs to double-brooding to better understand the value of double-brooding to lifetime fitness in this population.

## Methods

### Study system and field methods

Savannah sparrows are multi-brooded, migratory songbirds that breed throughout Canada and much of the northern United States, and winter in the southern United States, Mexico, and parts of Central America (Wheelwright and Mauck 1998; Wheelwright and Rising 2020). Females produce replacement nests upon the failure of the first brood and some attempt a second brood after successfully fledging their first (Wheelwright and Rising 2020). We refer to double-brooding as those situations where a female has successfully reared two broods to fledging. On Kent Island, 29% (range = 14–58% per year) of females attempted a second brood in a given year (27-year dataset; Woodworth et al. 2017). Pairs may be socially monogamous or polygynous (Wheelwright and Mauck 1998; Mueller et al. 2025). Polygynous mating in Savanah sparrows can reduce female fitness (Mueller et al. 2025), likely due to reduced male parental investment. Within females mated to polygynous males, non-primary females (females paired to a male after he already had a primary mate) had fewer fledglings and recruits than primary females (female paired to a male before he acquired a second mate; Mueller et al. 2025). Clutch sizes range from 2–6 per brood (median = 4), with second broods typically having smaller clutches than first broods (Wheelwright and Rising 2020). Young fledge ~9–11 days after hatching and parents provide post-fledging care for an average of 13 days after fledging (range 1–25 days; Wheelwright et al. 2003). On Kent Island, approximately 7–14% of nestlings banded on post-hatch day 7 will later recruit into the population as adults (Wheelwright and Rising 2020). Annual survivorship of adults on Kent Island varies across years (37–73%) and birds rarely live past 5–6 years of age (Wheelwright and Rising 2020).

**Fig. 1.**
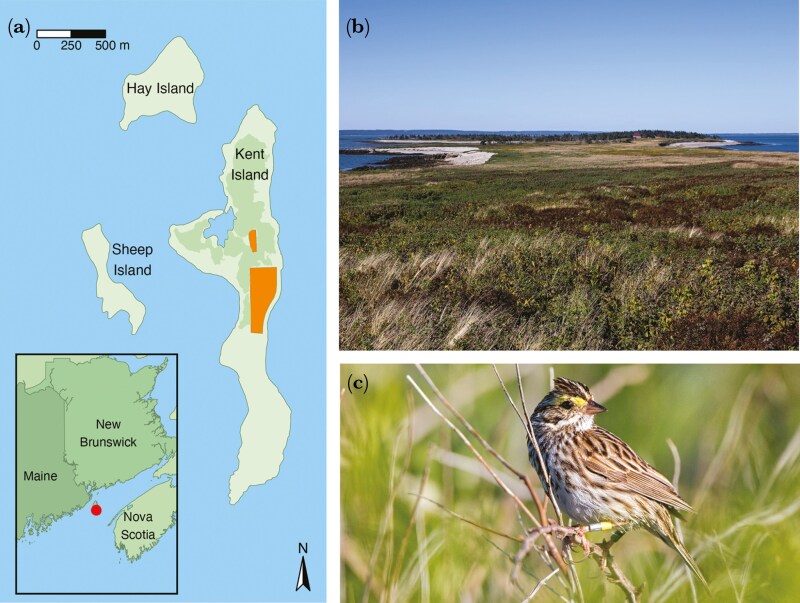
Study site and species: Savannah sparrows of Kent Island, New Brunswick. a) Map of the Three Islands (Kent, Hay, and Sheep Island). The map shows the location of the Three Islands in the Bay of Fundy, and the long-term Savannah sparrow study area (North Field and South Field, in orange). The lighter green areas of the Three Islands represent grassland breeding habitat for Savannah sparrows and the darker green area represents the area on Kent Island covered by spruce forests. Map by S.D.M. b) View of Kent Island in the Bay of Fundy from the south end. Photo by H.A.S. c) Photo of an adult Savannah sparrow. Photo by H.A.S.

We monitored Savannah sparrows annually on Kent Island (Fig. 1; 44.48° N, 66.79° W) from 1987 to 2023, excluding 2005-2007 and 2020. The study area consists of two open fields (North Field: 1.5 ha, and South Field: 6 ha) in the center of the island. Each season, new adults within the study area were banded with a unique combination of one United States Geological Survey/United States Fish & Wildlife Service/Canadian Wildlife Service (USGS/USFWS/CWS) aluminum leg-band and three coloured bands. Breeding partners were identified by observing social interactions between the pair within the territory. Nests were found by observing female incubation behaviors and, once found, were monitored every other day until hatching. In 2021 and 2022, a subset of females had their nests protected with predator exclosures (n = 25/35 females in 2021 and n = 32/33 females in 2022; these females were included in analyses; see below for justification). Nestlings were banded with a USGS/USFWS/CWS aluminum leg-band and one color band at 7 days post-hatch. To prevent premature fledging, nests were not visited following day-7 banding activities. Nests were monitored for fledging by observing parental defense or feeding behaviors following the 9^th^ day after hatching. After fledging of the first brood, we monitored territories of breeding pairs every 1-3 days to determine whether females would initiate a second brood. Single-brooded females produced one or more first nest attempts until a brood successfully fledged and then ceased breeding for the remainder of the season. Rarely, second broods failed owing to abandonment or predation (of the 294 females that attempted double-brooding, 10% [n = 30] failed to fledge any second brood offspring in the full dataset, half due to depredation and half due to abandonment). Of the females that attempted but failed double-brooding, 60% (18/30) failed during the incubation stage, and 40% (12/30) failed during the nestling stage. Because of differences in reproductive investment between failed double-brooders and successful double-brooders, as well as between failed double-brooders and single-brooders, we did not feel it was appropriate to group these individuals with either the single-brooded or successfully double-brooded individuals. Given the low sample size of individuals that attempted but failed double-brooding, we opted to remove these individuals from the dataset. All double-brooded females in the dataset successfully reared two broods to fledging.

### Data organization and statistics

To explore whether double-brooding poses costs to females or their offspring, we created four datasets. The first dataset included all females breeding between the years 1987 and 2022 (excluding the years when monitoring did not occur: 2005–2007 and 2020). We removed females breeding in 2004 and 2019 because survival and future breeding success could not be assessed due to the absence of monitoring in the following years. We also removed females for whom we were missing one or more nesting attempts and females that nested once or more outside the standard study area so that our dataset included all females for which a complete within-season breeding history was obtained (Woodworth et al. 2017). We also removed females whose nests were the subject of experimental manipulations (Woodworth et al. 2017). The first dataset was used for Models 1 and 2 (below). The second dataset included females that had breeding records for each year they were known to be alive within 1987–2022. Females that were known to have bred or had the potential to have bred in years when monitoring did not occur were removed from the dataset. We also removed birds that were still observed breeding in 2023, because we do not yet know their complete lifetime reproductive success. The second dataset was used for Model 3 (below). The third dataset included all F1 offspring hatched in the nests of females in dataset 1. We removed F1 offspring hatched between 2004-2007 and in 2019 (no monitoring 2005-2007 or 2020; recruitment is unknown) and in 2023 (recruitment not yet known). The third dataset was used for Model 4 (below). The fourth dataset included all recruited F1 female offspring from dataset 3 that met our inclusion criteria for dataset two. The fourth dataset was used for Models 5 and 6 (below). Statistics were conducted in R v4.2 (R Core Team 2023).

To test whether double-brooding influenced metrics of fitness, we used generalized linear mixed effects models (GLMMs; glmmTMB Package; Brooks et al. 2017). Individual models, including definitions for variables, are described below. Because rates of double-brooding in years with predator exclosures were higher than rates across some other years (Suppl. Figure 1), we tested whether including exclosure nests impacted results in Models 1, 2, and 4 (see *Model predictors* below; models where the dependent variable was not a measure of lifetime fitness). We found no substantive differences in the covariates included in the top models when we included the exclosure nests compared with when we excluded the exclosure nests. We therefore chose to include the exclosure nests in our models. For some models, we included covariates known to impact fitness in this population (see Suppl. Table 1 for details and justification). Each of these covariates (mating status, number of first-brood fledglings, hatch year population density, mother’s age, and mother’s mating status) could capture potential variance in additional challenges experienced during the breeding season. Thus, we included two-way interactions between our predictor variable related to double brooding and each relevant covariate (Table 1) to test the prediction that fitness trade-offs may only occur when double-brooding is combined with another energetically challenging situation. We used Pearson’s Product Moment Correlations to test for collinearities (p > 0.05) between covariates. We then built full models with all predictor variables and evaluated interactions suspected to be biologically important.

**Table 1.** **Model selection results for global models (Models 1–6).** Following model selection, we selected top models for each global model (ΔAIC_c_ < 2). The predictor variables in these top models are listed in the column “Top Model Predictors” and uninformative parameters are italicized. When top models differed by only one uninformative parameter, they were excluded from analysis (as indicated by “(excluded)” text within the “Top Model Predictors” column).

Global Model	Top Model Predictors	Log-likelihood	df	ΔAIC
**Model 1:** apparent survival ~ brood type × number of first brood fledglings + brood type × female mating status + (1|year) + (1| female ID), family = binomial	brood type × number of first brood fledglings	-673.87	6	0
	brood type + number of first brood fledglings	-675.47	5	1.17
**Model 2:** number of fledglings produced the following year ~ brood type × number of first brood fledglings + brood type × female mating status + (1| the following year) + (1| female ID) + intercept-only zero-inflation term, family = generalized Poisson	brood type × mating status	-968.36	10	0
	(excluded) brood type × mating status + n*umber of first brood fledglings*	-967.96	11	1.29
**Model 3:** lifetime recruitment ~ lifespan + number of successful seasons double-brooded × number of seasons mated to a polygynous male + number of successful seasons double-brooded × average population density across all years bred + (1| female’s hatch year), family = negative binomial	lifespan + number of successful seasons double-brooded	-403.78	6	0
	(excluded) lifespan + number of successful seasons double-brooded + *number of seasons mated to a polygynous male*	-403.70	7	1.90
**Model 4:** recruitment ~ natal brood number × hatch year population density + natal brood number × mother’s age + natal brood number × mother’s mating status + (1| offspring’s hatch year) + (1| mother’s ID/ nest ID), family = binomial	natal brood number	-1044.68	6	0
	(excluded) natal brood number + *mother’s age*	-1044.34	7	1.33
	(excluded) natal brood number + *mother’s mating status*	-1043.49	8	1.63
	(excluded) natal brood number + *annual density*	-1044.63	7	1.91
**Model 5:** lifetime fledging success ~ lifespan + number of successful double broods + natal brood number × hatch year population density + natal brood number × mother’s age at hatch year + natal brood number × mother’s mating status at hatch year + (1| offspring’s hatch year) + (1| mother’s ID) + intercept-only zero-inflation term, family = Poisson	lifespan + number of successful double broods	-207.75	6	0
**Model 6:** lifetime recruitment ~ lifespan + number of successful double broods + natal brood number × hatch year population density + natal brood number × mother’s age at hatch year + natal brood number × mother’s mating status at hatch year + (1| offspring’s hatch year) + (1| mother’s ID), family = Poisson	annual density + lifespan + number of successful double broods + natal brood number × mother’s age	-76.03	11	0
	annual density + lifespan	-83.94	5	0.79
	annual density + lifespan + natal brood number	-81.83	7	1.30
	annual density + lifespan + natal brood number × mother’s age	-78.13	10	1.52

We assessed all model combinations (MuMIn function: dredge; Bartoń 2023) and selected the top model(s) for each response variable (ΔAIC_c_ < 2; Burnham and Anderson 2002). We assessed each top model for uninformative parameters—parameters that occur in top models but there is no evidence for a relationship between this parameter and the response variable—and excluded top models if the only difference between that model and another top model was one additional uninformative parameter (Leroux 2019). When there were multiple top models, we used model averaging (MuMIn function: model.avg) and reported model averaged statistics for predictor variables. In all models, we determined 95% confidence intervals using the function confint() and we report the lower (2.5%) and upper (97.5%) limits for each parameter. We considered predictors to be important when the lower and upper confidence limits did not overlap with zero (Payton et al. 2003).

### Model predictors

We explored whether brood type (single- versus double-brooded) predicted apparent survival (breeding season to breeding season; Model 1) and the number of fledglings produced the following year (Model 2). Apparent survival was a two-level factor (0 = the female did not return in any future breeding season, 1 = the female did return in any future breeding season) and was modeled with a binomial distribution. The number of fledglings produced the following year was a continuous count variable and was modeled with a generalized Poisson distribution. In both Models 1 and 2, we included fixed effects of the interactions between brood type (two-level factor: 0 = single-brooded, 1 = double-brooded) and mating status (three-level factor: MO = female mated monogamously, PG1 = primary female of polygynous males, and PG2 = non-primary female of polygynous males) and between brood type and number of fledglings produced during the first brood (continuous count variable), as well as random effects of year and individual ID (both coded as factors; for additional details and justification, see explanations in Suppl. Table 1). We did not analyze paternity in this study and refer to the birds as either “monogamous” [or “socially monogamous”] when one female paired with one male, or “polygynous” when two females paired with one male. Given that mate-switching can occur between broods in this population mating status may not remain consistent across the season. We classified females as “PG2” when they were the non-primary female of polygynous males for one or more broods. We did not have any cases where females switched between having a monogamous mate and being the primary female of a polygynous mate between broods. To account for a higher number of nests that did not fledge offspring than would be predicted given a generalized Poisson distribution, an intercept-only zero-inflation term was added to Model 2. After detecting an important interaction between brood type and mating status, we calculated pairwise comparisons among the mating status groups using the emmeans package (emtrends function; Lenth 2023).

Our response variables of “apparent survival” and “recruitment” (below) reflect return rates. Philopatry levels are high on Kent Island, with ~11% of nestlings that were banded on post-hatch day 7 and ~45% of adults returning the following year (Wheelwright and Schultz 1994; Wheelwright and Mauck 1998). Furthermore, juveniles have not been observed to disperse more than 3 km away (no dispersal observed further than the Three Islands; Fig. 1) from their natal site and median dispersal distances are 228 m (~ six times the diameter of an average territory; Wheelwright and Mauck 1998). In a recent study, the detection probability of Savannah sparrows within the study site on Kent Island was high: out of 11,175 individuals reported between 1987 to 2022, only 113 individuals were not observed for one or more years between detections (~1% of individuals; Mueller et al. 2025). Therefore, we did not use capture–recapture analysis for survival or recruitment and assumed that individuals not returning to Kent Island had died rather than dispersed.

We explored whether the number of successful seasons double-brooding predicted lifetime recruit production (F1 offspring production; Model 3). Lifetime recruit production was a continuous count variable measuring the sum of the number of F1 recruits (number of offspring returning to breed on the island following their hatch year) produced by a female across her lifespan. Lifetime recruit production was modeled with a negative binomial distribution. In this model, we included fixed effects of lifespan (continuous count variable: last year the individual was observed on Kent Island subtracting the individual’s hatch year) and two-way interactions between the number of times females were mated to a polygynous mate during her lifetime (continuous count variable) and the number of successful double broods (three-level factor: 0 = females that never double-brooded; 1 = females that double-brooded once; and 2 = females that double-brooded twice or more across their lifespan), as well as between average population density across all years bred (population density was measured as the peak number of breeding adults of both sexes in the study area in each year; Woodworth et al. 2017) and the number of successful double broods (for additional details and justification, see explanations in Suppl. Table 1). We included mother’s hatch year as a random effect (Suppl. Table 1).

We examined whether F1 offspring survival to their first breeding season (offspring recruitment) differed between offspring of single-brooded mothers, first-brood offspring of double-brooded mothers, or second-brood offspring of double-brooded mothers (hereafter, referred as “natal brood number”; Model 4). Two individuals with unknown hatch dates were excluded from the dataset. F1 offspring recruitment was a two-level factor (0 = offspring that did not return to Kent or neighboring islands (Fig. 1) after their first winter, and 1 = offspring that were observed on Kent or neighboring islands in any year following their hatch year). F1 offspring recruitment was modeled with a binomial distribution. We included two-way interactions between natal brood number (three-level factor: 1 = F1 offspring hatched to single-brooded females, i.e. single brood offspring, 2 = F1 offspring hatched to first broods of double-brooded females, i.e. first brood offspring, and 3 = F1 offspring hatched to second broods of double-brooded females, i.e. second brood offspring) and hatch year population density (continuous count variable), mother’s age (two-level factor: 0 = females one year of age, i.e. second-year females, and 1 = females two years of age or older, i.e. after-second-year females), and mother’s mating status (three-level factor; see above) as fixed effects, as well as random effects of hatch year and natal nest ID nested in mother’s ID (for additional details and justification, see explanations in Suppl. Table 1). We also describe a correlation between natal brood number and hatch date and discuss possible implications.

We examined whether natal brood number predicted lifetime fledging success of F1s (F2 fledglings produced; Model 5) and lifetime recruitment of F1s (F2 recruits produced; Model 6). Lifetime fledgling success (the total number of F2 fledglings produced by an F1 female across their lifespan; fledglings were defined as the number of offspring surviving to post-hatch day 7) and lifetime recruit success (the total number of F2 recruits produced by an F1 female across their lifetime) were continuous count variables. Both response variables were modeled with a Poisson distribution. In both models, we included fixed effects of lifespan and interactions between natal brood number and hatch year population density, mother’s age in hatch year, and mother’s mating status in hatch year, as well as random effects of hatch year and mother’s ID (for additional details and justification, see explanations in Suppl. Table 1). Again, to account for a higher number of nests that did not fledge offspring than would be predicted by a Poisson distribution, both models included an intercept-only zero-inflation term.

## Results

### Female Fitness

Two top models best predicted apparent survival of females to the following season (Model 1; ΔAICc < 2; Table 1). After model averaging, there was evidence that survival differed between single- and double-brooded females (𝛽 = 0.33, *z* = 2.16, 95% CI = 0.03, 0.63; Suppl. Figure 2). Approximately 55% (149/273) of double-brooded females returned compared with 46% (329/709) of single-brooded females. Of the females that attempted but failed double-brooding (not included in analysis), 33% (10/30) returned. Females that had more fledglings in their first broods were more likely to survive (𝛽 = 0.12, *z* = 2.30, 95% CI = 0.02, 0.22) than females that had fewer fledglings in their first broods. There was no evidence that the interaction between brood type and the number of first-brood fledglings predicted survival (𝛽 = -0.16, *z* = 0.98, 95% CI = -0.49, 0.17).

**Fig. 2.**
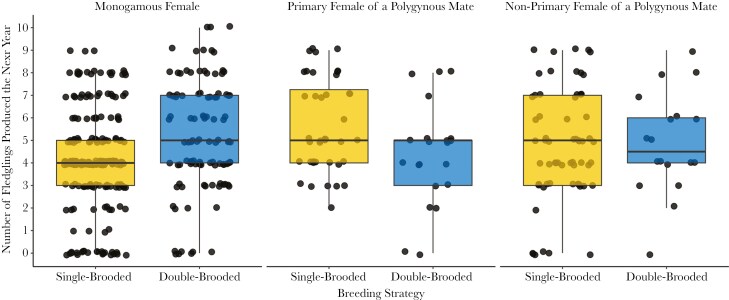
Next year F1 fledgling production in single- versus double-brooded female Savannah sparrows based on the previous year’s breeding histories. Within monogamous females, double-brooded females produced more F1 fledglings the following year than single-brooded females. There was no evidence for differences in F1 fledgling production the following year between single- and double-brooded primary females from polygynous groups or non-primary females from polygynous groups. The boxes show the 25th to 75th percentile of data, with the mean shown by the thicker black bar. Error bars represent 1.5x interquartile range.

There were two top models that best predicted the number of fledglings produced by females the following year (Model 2) but the second top model only differed from the first by the inclusion of one uninformative parameter and, thus, was not considered (Table 1; Leroux 2019). Pairwise comparisons revealed that, for socially monogamous females (n = 312 females), double-brooded females produced more fledglings the following year than single-brooded females (𝛽 = 0.18, *z* = 3.52, 95% CI = 0.03, 0.33; Fig. 2). For primary females from polygynous groups (n = 61) and non-primary females from polygynous groups (n = 80), there was no difference in the number of fledglings produced the following year between single- and double-brooded females (primary females: 𝛽 = -0.17, *z* = -1.62, 95% CI = -0.13, 0.46; non-primary females: 𝛽 = -0.02, *z* = -0.22, 95% CI = -0.26, 0.31).

**Fig. 3.**
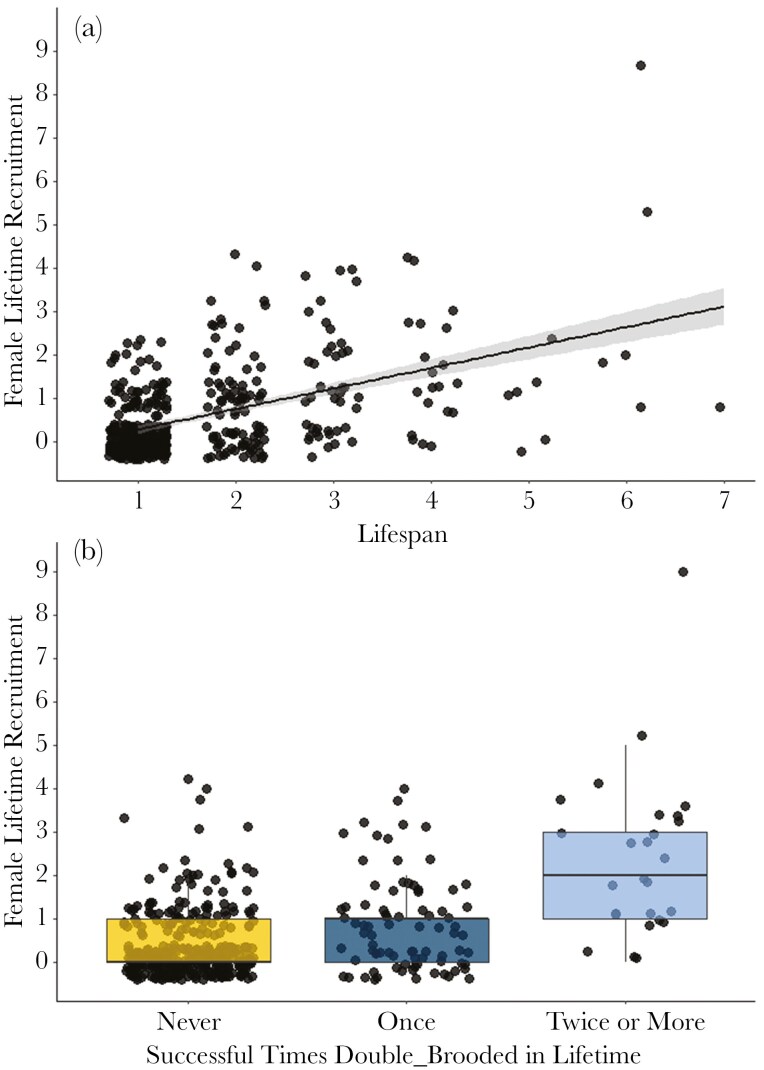
Relationship between (a) lifespan and (b) the number of times double-brooding and lifetime recruit production in female Savannah sparrows. The number of successful second broods was positively related to lifetime recruit production (F1 recruits produced) in a model that included lifespan as a covariate. Females with longer lifespans had higher F1 lifetime recruit production and females that double-brooded once and females that double-brooded twice or more had higher F1 lifetime recruit production than females that never double-brooded. The boxes show the 25th to 75th percentile of data, with the mean shown by the thicker black bar. Error bars represent 1.5x interquartile range.

There were two top models that best predicted lifetime recruit production (number of F1 offspring females produced within their lifetime; Model 3) but, similar to Model 2, one of these top models contained an uninformative parameter and was not considered (Table 1). The top model demonstrated that the number of F1 recruits increased with lifespan (𝛽 = 0.31, *z* = 5.24, 95% CI = 0.20, 0.43) and that, compared with females that never double-brooded, females that were double-brooded once (n = 83, 𝛽 = 0.53, *z* = 3.00, 95% CI = 0.18, 0.88) or twice or more (n = 26, 𝛽 = 0.96, *z* = 3.81, 95% CI = 0.47, 1.46) produced more recruits over their lifetime (n = 334; Fig. 3).

### F1 offspring fitness

There were four top models that best predicted recruitment of F1 offspring (Model 4) but, as with Models 2 and 3, three of these top models contained uninformative parameters and were not considered (Table 1). The top model demonstrated that F1 offspring hatched in first broods of double-brooded females had greater recruitment than F1 offspring hatched in second broods (𝛽 = 0.66, z = 4.27, 95% CI =0.36, 0.96) and F1 offspring hatched in single broods (Suppl. Figure 3; 𝛽 = 0.41, z = 2.58, 95% CI =0.10, 0.72). There was no evidence that recruitment differed between F1 offspring hatched in single broods and F1 offspring hatched in second broods of double-brooded females (𝛽 = 0.25, z = 1.43, 95% CI = -0.09, 0.60). Approximately 17% (162/970) of F1 offspring hatched in first broods recruited, 12% (112/968) of F1 offspring hatched in single broods recruited, and 10% (n = 86/869) of F1 offspring hatched in second broods recruited. Natal brood number was highly correlated with hatch dates (Pearson’s product moment correlation: r = 0.84, df = 2805, t = 83.55, p < 0.001). Mean hatch dates for single broods, first broods, and second broods were June 15 (ordinal date 166; range 150-191), June 11 (ordinal date 162; range 150-179), and July 13 (ordinal date 194; range 179-212), respectively.

**Fig. 4.**
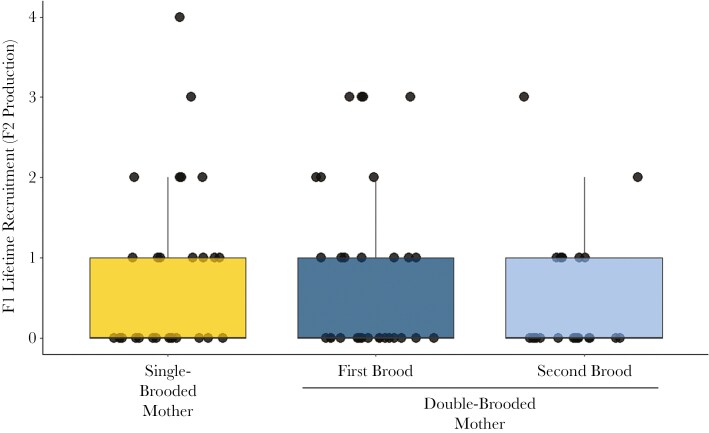
Lifetime recruitment (F2 recruits produced) across natal brood numbers in female F1 offspring of single- and double-brooded Savannah sparrows. Lifetime recruitment did not differ between F1 offspring hatched in single broods, F1 offspring hatched in first broods, and F1 offspring hatched in second broods. Error bars represent 1.5 x interquartile range.

The top model predicting lifetime fledgling production (number of F2 fledglings produced by F1 female offspring within their lifetime; Model 5) included lifespan and the number of double broods produced (Table 1). The number of lifetime F2 fledglings increased with lifespan (𝛽 = 0.28, z = 6.42, 95% CI =0.19, 0.36) and with the number of double broods produced (𝛽 = 0.30, z = 2.64, 95% CI =0.08, 0.53). There were four top models that best predicted lifetime recruit production (number of F2 recruits produced by F1 female offspring within their lifetime; Model 6; Table 1). Model averaging suggested that lifespan was positively related to lifetime F2 recruit production (𝛽 = 0.33, z = 3.36, 95% CI =0.14, 0.53) and hatch year annual density was negatively related to lifetime recruit production (𝛽 = -0.03, z = 3.34, 95% CI = -0.05, -0.01). After model averaging, there was no evidence that having a double-brooded mother influenced lifetime F2 recruitment: lifetime F2 recruitment did not differ between first- and second-brood offspring (n = 31 first-brood offspring; n = 23 second-brood offspring, 𝛽 = 0.22, z = 0.27, 95% CI = -1.36, 1.86), first- and single-brood offspring (n = 27 single-brood offspring; 𝛽 = 0.44, z = 0.60, 95% CI =-1.88, 0.99), or second- and single-brood offspring (Fig. 4; 𝛽 = -0.22, z = 0.50, 95% CI = -1.09, 0.64). The was also no evidence for an effect of the number of double broods produced (𝛽 = 0.15, z = 0.66, 95% CI = -0.30, 0.60) or for an interaction between natal brood number and mother’s age at hatching (all comparisons 95% confidence limits overlapped with zero).

## Discussion

Through a 30-year field study of reproductive success in wild, migratory Savannah Sparrows, we discovered there were fitness benefits to double-brooding and no apparent trade-offs between double-brooding and female survival or future reproductive success. We also showed that, while second-brood F1 offspring had lower recruitment than first- and single-brood F1 offspring, 10% of second-brood F1 offspring did recruit, leading to double-brooded females having higher lifetime recruitment rates than single-brooded females. To the best of our knowledge, we also provide the first comparison of the number of lifetime recruits produced by offspring hatched in nests of single- versus double-brooded females, revealing no significant differences in F2 recruits from F1 offspring arising from double-brooded females than F2 recruits from F1 offspring arising from single-brooded females. Our research demonstrates that double-brooding is a highly beneficial reproductive strategy for Savannah sparrows and females that can double-brood do so with no detectable net cost to fitness.

Life history theory posits that a breeding attempt will occur when the expected benefits of breeding outweigh anticipated costs (Stearns 1976). Despite the benefits of double-brooding to lifetime fitness in this population, annual double-brooding rates in Kent Island Savannah sparrows averaged only 29% (range:14-58%) over 27 years (Woodworth et al. 2017). If double-brooding is such a profitable breeding strategy, why do so many individuals remain single-brooded? The timing of breeding (annual first laying date) and nest predation were two factors that significantly predicted double-brooding rates in this population (Woodworth et al. 2017). In other words, females that nested early had a higher probability of double brooding and, if females had their first nest depredated, they rarely double-brooded (2% [7/315] of females that lost their first nest attempt successfully double-brooded; Woodworth et al. 2017). However, even among those females that successfully fledged offspring from their first nest attempt, only 56% initiated second broods (Woodworth et al. 2017). Moreover, in 2022 when 41 pairs had their first nest attempts protected by predator exclosures (Spina et al. 2025), 15 females did not attempt second broods. Of those females that fledged their first nest attempt, timing of breeding was negatively related to the probability of double-brooding (Woodworth et al. 2017). Female age and local population density also influenced double-brooding probabilities where older females from lower density populations were more likely to double brood (Woodworth et al. 2017). Double-brooding was moderately repeatable in our population (~10% of the variance in double-brooding using dataset 1 is explained by individual using rpt analysis; Stoffel et al. 2017). Although previous studies have supported the hypothesis that female quality can influence the probability of double-brooding (Cornell and Williams 2016), it is notable that the full model in Woodworth et al. (2017) did not include a measure of individual quality.

Our findings that double-brooded females did not exhibit a trade-off between current and future fitness suggests that double-brooded females may be high-quality individuals. Positive correlations between survival and reproductive success can be observed when there is variation in individual quality within a population (van Noordwijk and de Jong 1986). High-quality individuals, with access to a greater pool of resources compared with lower-quality individuals, can allocate these resources toward multiple life-history traits, achieving high reproductive success while avoiding costs to survival or future fitness (The Quality Hypothesis; van Noordwijk and de Jong 1986; Stouffer 1991; Reznick et al. 2000; Mitchell et al. 2012). A recent study on this population demonstrated that females with lower baseline corticosterone levels during the first brood nestling period had higher body condition and fat scores and a higher probability of double-brooding that season, supporting the Cort-Fitness Hypothesis (i.e. higher levels of baseline corticosterone reflect poor condition and reduced fitness; Bonier et al. 2009). Corticosterone is considered a metabolic hormone that increases with energetic needs associated with environmental challenges (Bonier et al. 2009). The finding that double-brooded females had lower corticosterone levels near the end of the first brood nestling period suggests that double-brooded females were able to successfully rear a first brood while maintaining a lower metabolic load than single-brooded females. Thus, lower baseline corticosterone in double-brooded females during the first brood nestling phase suggests a positive relationship between female quality and double-brooding (Spina et al. 2025). Females that double-brood in this population achieve high reproductive success without costs to apparent survival, which provides further support for the hypothesis that maternal quality influences the probability of double-brooding. Future research exploring factors associated with maternal quality could provide a stronger understanding of low double-brooding rates. For example, studies that investigate variation in resource acquisition (e.g. foraging efficiency, diet quality, or nutrient retention) in single- and double-brooded parents could explore this question.

Although we did not find evidence for costs of double-brooding, our results demonstrated that double-brooded females produced more F1 fledglings the following year than single-brooded females only when they were paired with a monogamous mate, rather than a polygynous mate, during their first brood. Because there were no differences in the number of F1 fledglings produced the following year within females that were paired to polygynous males, double-brooded females may only demonstrate higher reproductive success the following year when paired monogamously. The potential combined cost of being double-brooded and mated to a polygynous male the following year provides an interesting topic for future study; comparing metrics of female condition (e.g. fat scores, corticosterone levels, telomere lengths, oxidative stress) at the end of the breeding season in females in each brood type–by–mating status group could provide insight into the combined energetic costs of double-brooding and being a part of a polygynous group.

We found that hatch year density was negatively related to lifetime F2 recruitment, an interesting result which suggests that high density conditions experienced by F1 females during early life may provide a more challenging early life environment that carries-over to influence offspring survival. Under high density conditions, more energy may be spent navigating competition with conspecifics for food, mates, or territories. For example, in Black-throated Blue Warblers (*Dendroica caerulescens*), experimentally reduced density was associated with increased time spent foraging and less time spent on territorial defense and mate guarding (Sillett et al. 2004). Additionally, on Kent Island, higher Savannah sparrow density has been associated with higher levels of predation (Woodworth et al. 2017), which could potentially increase stress (Scheuerlein et al. 2001). Thus, we hypothesize that the hatch year density could affect offspring survival because high density conditions provide a more challenging early life environment, which can carry-over to impact later life reproductive success. Future research in this population using predator exclosures to prevent nest predation and investigating the impacts of density on Savannah sparrow adult fitness and offspring development will be a valuable contribution.

Neither having a mother that was mated to a polygynous mate nor having a mother that was in her first breeding season impacted F1 offspring recruitment or lifetime reproductive success of F1 females. Given that females in their first breeding season have reduced breeding success—first breeding season females were more likely to initiate breeding later and have their first nest depredated—compared with females in their second or later breeding seasons (Woodworth et al. 2017), offspring of first year breeders may have a more challenging early-life environment. However, these early-life effects did not translate to differences in F2 recruitment. Because neither being mated to a polygynous mate nor being a first-time breeder influenced offspring fitness, these scenarios may not pose as substantially challenging situations for nestlings.

Among the F1 offspring that survived their first winter, differences in annual or lifetime reproductive success were not observed between offspring of single- versus double-brooding mothers, suggesting no net multigenerational costs of double-brooding. Moreover, first-brood F1 offspring were more likely to recruit than F1 single-brood offspring, suggesting that double-brooded parents are not likely trading-off first brood post-fledging care to begin second broods. In this population, the length of the interbrood interval did not influence first brood offspring recruitment, suggesting that double-brooded parents are able to adequately care for first brood fledglings, even when second broods are initiated soon after fledging (Spina et al. 2025). Multigenerational costs to double-brooding could occur if double-brooding results in poor developmental conditions for offspring that carry-over to influence later life fitness. In the Kent Island Savannah sparrows, nestling weight at day 7 predicted pre-migratory condition and recruitment (Mitchell et al. 2011) and because nestling mass was lower in second broods (unpublished data), this relationship may explain why fewer F1 second-brood nestlings recruited. Our results suggest that conditions experienced during early-life may impact survival, but if offspring are able to recruit, they do not experience the impact of early-life growth rate on later-life reproductive success (i.e. the number of offspring produced).

Our study provides one of the few assessments of the long-term fitness outcomes of double-brooding in a ground-nesting species. Previous studies examining whether double-brooding impacted lifetime fledging success or recruitment were conducted in southern house wrens (*Troglodytes aedon musculus*; Carro et al. 2014), Eurasian hoopoe (*Upupa epops*; Hoffmann et al. 2015), Cassin’s auklets (*Ptychoramphus aleuticus*; Johns et al. 2018), barn owls (*Tyto alba*; Zabala et al. 2020), sedge warblers (*Acrocephalus schoenobaenus*; Zając et al. 2015). Over half to all individuals within these populations, except the sedge warblers (Zając et al. 2015), built their nests within nest boxes (Carro et al. 2014; Hoffmann et al. 2015; Johns et al. 2018; ; Zabala et al. 2020). Factors influencing the energetic demand of rearing offspring, such as predation risk, abiotic conditions experienced at the nest, and whether nests are reused could be altered by the use of nest boxes compared with natural cavities and ground nests. These differences in nest type and their implications for the costs and benefits of double brooding may partly explain some of the discrepancies between our results (from a ground-nesting species) and those of previous studies.

Overall, our study provides thorough assessment of the multigenerational costs and benefits of double-brooding in female Savannah sparrows. Despite increasing lifetime reproductive success, generally we did not detect any net long-term costs to double-brooding, likely because the costs of double-brooding may be mitigated by high quality (Stouffer 1991; Mitchell et al. 2012). Some studies have shown that costs to double-brooding can emerge only in cases where additional stressors interact with the increased energetic load of rearing two broods (Verhulst 1998; Saino et al. 1999), but we did not find evidence that costs of double brooding emerged when double-brooding coincided with other potentially challenging activities, including rearing more first-brood F1 offspring, higher population density, or having less breeding experience. However, we did detect a potential combined cost of being double-brooded and being mated to a polygynous male in one year on the number of fledglings produced the following year, which provides an interesting topic for exploration in future studies. While our findings do not negate the possibility of hidden costs not yet explored—for example, epigenetic effects induced by parental or environmental programming could emerge in F3s (Harney et al. 2022; Tando and Matsui 2023)—given the lack of net costs to double-brooding, the emergence of substantial hidden costs that result in trade-offs to future reproductive success remain unlikely. This study demonstrates the high value of double-brooding to fitness in a migratory songbird, with no multigenerational net costs on lifetime fitness.

## Supplementary Material

araf040_suppl_Supplementary_Materials_1

## Data Availability

Analyses reported in this article can be reproduced using the data provided by Spina et al (2025).
