# Supplementary material for: Multigenerational fitness outcomes of double-brooding: a 30-year study of a migratory songbird
Source: Behav Ecol. 2025 May 4;36(4):araf040. doi: 10.1093/beheco/araf040 (PMC12137901; doi:10.1093/beheco/araf040)
Supplement: araf040_suppl_Supplementary_Materials_1 [file araf040_suppl_supplementary_materials_1.docx]

### **Supplemental Information**

**Figures**


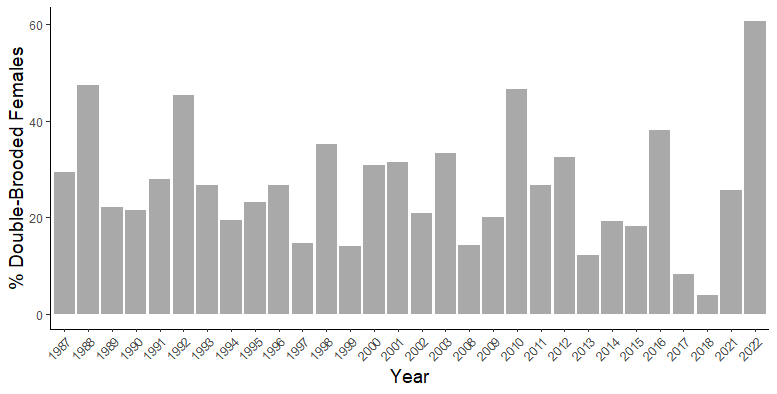


**Suppl. Figure 1. Rates of successful double-brooding (rearing two broods to fledging) in female Savannah sparrows from 1987 - 2022.** Double-brooding rates were variable across years (range 3.8% - 60.6%). Double-brooding rates in years with predator exclosures (2021, 2022) were higher than some other years. Some years are excluded due to the absence of monitoring (2005-2007, 2020) or because all females in those years were excluded from analyses (2004, 2019; see *data organization and statistics* for details).


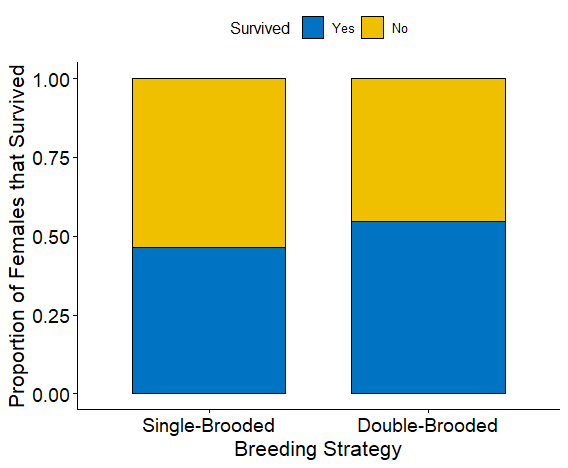


Suppl. Figure 2. Apparent survival of single- versus double-brooded female Savannah sparrows. Return rates (a proxy for survival) were higher in double-brooded females compared to single-brooded females. Approximately 55% of double-brooded females returned (n = 149/273) compared to 46% of females that reared only one brood (n = 329/709).


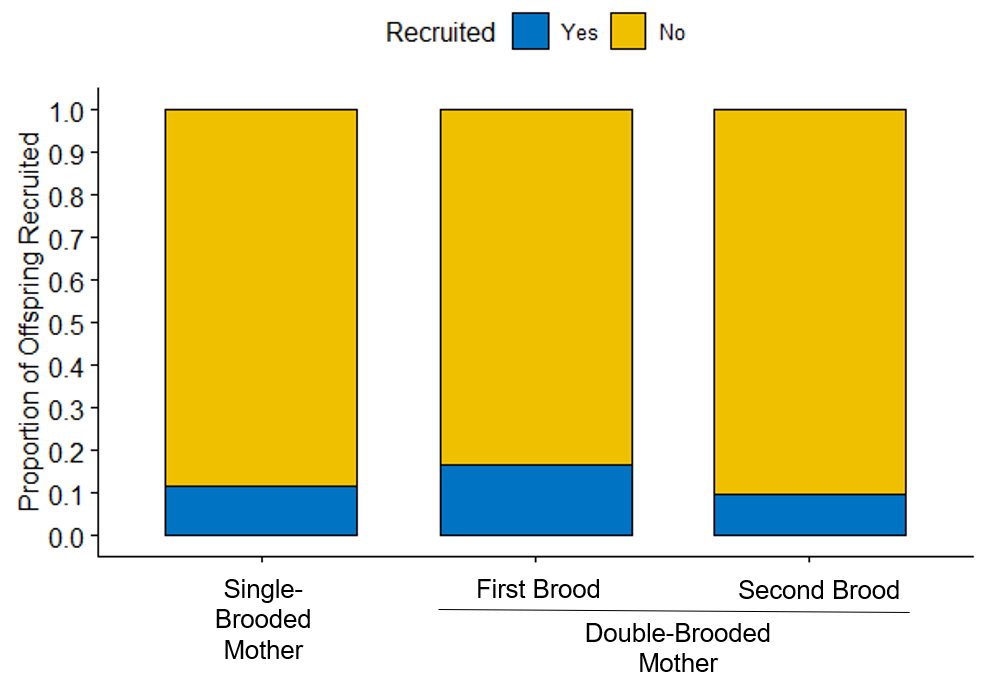


Suppl. Figure 3. Recruitment differences across natal brood numbers in female offspring of single- and double-brooded Savannah sparrows. Recruitment was higher in offspring hatched to first broods compared to single broods and second broods. Recruitment did not differ between offspring hatched to second broods and offspring hatched to single broods.

Tables

Suppl. Table 1. Additional variables included in models. In addition to fixed effects related to double-brooding, the following fixed and random effects were included as additional variables in our models. Rationale is provided.

| **Models** | **Variable and type of predictor** | **Rationale for inclusion** |
| --- | --- | --- |
| 1 – 2 | Number of first brood fledglings; fixed effect | In Mueller et al. (*in review*), there was evidence that survival in this population was predicted by the total number of fledglings produced. However, in our dataset, this predictor was highly correlated with brood type (Pearson’s product-moment correlation: r = 0.75). We, therefore, tested whether the number of fledglings produced during the first brood was correlated with brood type because this variable could also reflect parental investment (i.e. raising more first brood offspring in a season could be considered a challenging scenario; see Suppl. Table 4.2 for explanation). The number of fledglings produced during the first brood was not correlated with brood type (r = 0.14). |
|  | Mating status; fixed effect | In Mueller et al. (*in review*), there was evidence that survival in this population was predicted by an interaction between mating status (being mated to a monogamous compared to a polygynous mate) and female age. However, in our dataset, female age was correlated with brood type (Pearson’s product-moment correlation: r = 0.28). Therefore, we included mating status, but not an interaction with female age, in these models. |
|  | Year; random effect | To account for among-year differences in environmental conditions that could have influenced survival or fecundity. |
|  | Individual ID; random effect | To account for repeated sampling of females across years. |
| 3, 5 – 6 | Lifespan; fixed effect | To control for the number of seasons a female was recorded breeding. |
| 3 | Number of times females were mated to a polygynous mate during her lifetime, average population density across all years bred; fixed effects | In Mueller et al. (*in review*), there was weak evidence that that mating status (monogamous or polygynous) and population density predicted offspring recruitment. Mueller et al., (*in review*) found that fledge date additionally influenced recruitment, but we did not include fledge date in our model because fledge date was highly correlated with natal brood number (r = 0.84). Mueller et al., (in review) and Mitchell et al., (2011) also found that nestling weight at 7-days post-hatching influenced recruitment, but we did not include nestling weight in our models because this variable was also weakly correlated with natal brood number (r = -0.20) and because we were missing these data from many individuals (n = 770/2908 individuals had no weight data). Mueller et al., (*in review*) also found that parental age influenced recruitment, but we did not include this variable in our models (see above Mating Status for explanation). |
|  | Mother’s hatch year; random effect | To account for cohort effects. |
| 4, 5 – 6 | Hatch year population density, mother’s age in hatch year, and mother’s mating status in hatch year; fixed effects | These predictor variables were previously demonstrated to influence recruitment in this population (Mueller et al., in review). |
|  | Hatch year | To account for among-year differences in environmental conditions that could have influenced offspring development and future fitness. |
| 4 | Natal nest ID nested in mother’s ID; random effect | To account for the non-independence of siblings that shared a nest environment and/or were reared by the same mother. |
| 5 – 6 | Mother’s ID | See above Natal nest ID nested in mother’s ID for explanation. We did not include natal nest ID in these models because no individuals hatched in the same nest. |
